# Leveraging Machine Learning for Porosity Prediction in AM Using FDM for Pretrained Models and Process Development

**DOI:** 10.3390/ma18194499

**Published:** 2025-09-27

**Authors:** Khadija Ouajjani, James E. Steck, Gerardo Olivares

**Affiliations:** 1 National Institute for Aviation Research, Wichita, KS 67260, USA; gerardo.olivares@idp.wichita.edu; 2 Department of Aerospace Engineering, Wichita State University, Wichita, KS 67260, USA; james.steck@wichita.edu

**Keywords:** porosity, defect prediction, fused deposition modeling (FDM), machine learning (ML), convolutional neural networks (CNN), multi-layer perceptron (MLP), additive manufacturing (AM), data-driven approach

## Abstract

Additive manufacturing involves numerous independent parameters, often leading to inconsistent print quality and necessitating costly trial-and-error approaches to optimize input variables. Machine learning offers a solution to this non-linear problem by predicting optimal printing parameters from a minimal set of experiments. Using Fused Deposition Modeling (FDM) as a case study, this work develops a machine learning-powered process to predict porosity defects. Specimens in two geometrical scales were 3D-printed and CT-scanned, yielding raw datasets of grayscale images. A machine learning image classifier was trained on the small-cube dataset (~2200 images) to distinguish exploitable images from defective ones, averaging over 97% accuracy and correctly classifying more than 90% of the large-cube exploitable images. The developed preprocessing scripts extracted porosity features from the exploitable images. A repeatability study analyzed three replicate specimens printed under identical conditions, and quantified the intrinsic process variability, showing an average porosity standard deviation of 0.47% and defining an uncertainty zone for quality control. A multi-layer perceptron (MLP) was independently trained on 1709 data points derived from the small-cube dataset and 3746 data points derived from the large-cube dataset. Its accuracy was 54.4% for the small cube and increased to 77.6% with the large-cube dataset, due to the larger sample size. A rigorous grouped k-fold cross-validation protocol, relying on splitting data per cube, strengthened the ML algorithms against data leakage and overfitting. Finally, a dimensional scalability study further assessed the use of the pipeline for the large-cube dataset and established the impact of geometrical scaling on defect formation and prediction in 3D-printed parts.

## 1. Introduction

Additive manufacturing (AM) offers significant advantages in design complexity and flexibility, driving its adoption in aerospace, automotive, and other industries seeking lightweight and intricate components. However, persistent challenges in repeatability, variable mechanical properties, and quality control continue to hinder certification and large-scale production.

In general, developing an optimal AM build requires generating comprehensive datasets and often entails resource-intensive trial-and-error. In particular, for costly processes such as powder bed fusion (PBF), material consumption, operational demands, and the need for an extensive multiscale characterization result in expensive experimental studies and limit the number of repetitions, the investigated scale, and the quantity of coupons that can be produced without major defects. Fused Deposition Modeling (FDM), though based on different material and process physics, exhibits analogous porosity defects (e.g., inter-bead gaps or intra-layer voids) to those observed in PBF. This morphological similarity positions FDM as a potential proxy for the initial study of defect formation and for validating machine learning pipelines for layer-wise prediction. While inferences from the study cannot be directly extended to predict the mechanical performance of PBF-produced parts, this work instead seeks to establish and validate a pipeline for defect types arising from process variability using ML. Because data structures and patterns associated with flaw detection are often agnostic to the specific material system, the focus here is on the geometry, distribution, and topological description of porosity.

FDM illustrates both the potential and limitations of AM. Its affordability and ease of use enable the rapid production of complex designs. The process involves depositing molten thermoplastic beads onto a heated bed, guided by CAD-derived toolpaths, with bead thickness (0.1–0.8 mm) determined by nozzle diameter. However, as with other AM processes, the quality of FDM parts is highly sensitive to input variables, with porosity emerging as an internal defect that critically undermines mechanical performance and hinders certification viability. The existing guidelines define allowable defect levels based on application-specific requirements [1]. The relationships between porosity, mechanical properties, and process parameters remain poorly understood.

The quality of FDM outputs is governed by numerous variables that interact without established physical laws and significantly influence performance. Kechagias et al. [2] reviewed experimental studies on the impact of process parameters on 3D-printed parts: most investigations considered only a few printing variables, were limited to identifying optimal parameter sets through batches of test coupons, and evaluated quality primarily at the coupon level. Few studies addressed numerical optimization, and those focusing on a single part’s characteristics did not yield solutions that could be realistically implemented at an industrial scale. Moreover, not all FDM printers provide full access to or a complete range of process parameters. While considering all parameters and running every combination would be a rigorous strategy, it remains prohibitively time- and cost-intensive. In addition, focusing only on coupon-level builds fails to capture the impact of process parameters on the anisotropic structure of printed parts. Kristiawan et al. [3] further reviewed the factors and input parameters affecting the quality and mechanical properties of FDM parts. They concluded that the relationships between process parameters remain poorly understood, ongoing investigations lack conclusive analytical models, and multiple interlinking variables can either mitigate or exacerbate defects in the resulting parts.

Most current research on FDM focuses on the impact of printing process parameters on the properties of printed parts. Chacón et al. [4] studied the effect of build orientation, layer thickness, and feed rate on the mechanical properties of PLA samples manufactured with a low-cost desktop 3D printer. Because only scattered input values were tested, the results were relevant only to the specific range of variables used. Using a design-of-experiments scheme and focusing on five main parameters, Gebisa et al. [5] found that raster angle and raster width strongly influenced the flexural properties of 3D-printed parts made of ULTEM 9085. However, their results cannot be generalized to other raw materials or mechanical properties, since they rely on a selective subset of input values. Similarly, Durgun et al. [6] investigated different raster angles for principal part orientations and tested specimens for surface roughness, tensile, and flexural strength. Rankouhi et al. [7] examined multiple prints and correlated the elastic modulus and ultimate strength with layer thickness. Their results are useful for decision-making when layer thickness is the key parameter. Other studies have examined printer brands (Stratasys, HP, and EOS) [8], infill percentages [9], and material/temperature effects [10,11], but such results lack generalization due to non-linear parameter interactions and dataset specificity. Conventional physics-based models struggle to capture all these interplaying dynamics, underscoring the need for data-driven approaches.

Machine learning (ML) addresses these gaps by generalizing non-linear functions from limited data and has become a widely used predictive and classification tool in AM. Several studies have reported promising results in materials science for property prediction [12], design [13], and smart manufacturing [14], reducing reliance on costly experimentation. ML methods such as kernel ridge regression have been used to map a material’s fingerprint with its known properties [14]. Frameworks have been developed to predict the properties of a wide variety of materials using decision trees [15] or the wear timeline of tools and the maintenance requirements using different types of neural networks [16,17,18].

**Figure 1:**
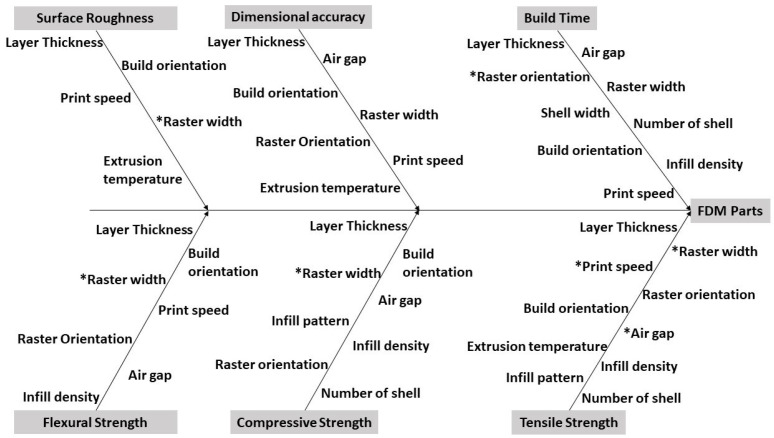
Main input variables impacting FDM print outcome (the impact of properties marked with * is unknown) [34] (available via CC BY).

FDM defects, in particular porosity, are, therefore, strong candidates for ML investigations, given the number of process parameters and the interactions between them (e.g., print speed, infill density, and temperature; see Figure 1). The existing parameter optimization methods—such as Taguchi designs [19,20,21,22], genetic algorithms [23], and hybrid approaches [24]—focus on dimensional accuracy [25,26,27,28] or mechanical performance [29,30,31]. However, defect prediction, especially porosity, currently relies heavily on statistical models focused on volumetric data (e.g., ANOVA and response surface methodology [32]) or combinatorial algorithms (e.g., BES and PSO [33]), which are limited by experimental scope and material heterogeneity, and do not give insight into the layer-wise buildup of porosity.

For defect investigation, machine learning combined with computed tomography (CT) has become a powerful approach for analyzing porosity [35]. Most published studies to date focus on metal AM, with few directly addressing FDM. For example, Pak et al. [36] applied deep convolutional models to map thermal imaging streams to CT-measured porosity in laser powder bed fusion (LPBF) parts, demonstrating that CNN architectures can localize and quantify porosity with high fidelity against CT data. Similarly, Mohammed et al. [37] trained neural networks directly on CT volumes to predict porosity distribution in LPBF specimens. Wang et al. [38] further introduced a defect-driven, physics-informed neural network framework that embeds melt-pool physics into the learning process, producing more physically consistent defect predictions than black-box ML. In contrast, for FDM processes, published ML studies on internal defects are scarce and have concentrated on in situ anomaly detection rather than CT-based porosity mapping. For instance, Goh et al. [39] implemented real-time ML anomaly detection in fused filament fabrication using thermal and visual sensors, underscoring the feasibility of applying image-based ML for internal defect prediction.

Investigations in metal AM validate the capability of ML architectures—including CNNs, MLPs, and physics-informed models—to predict defects from both imaging and process data. Building on this foundation, the present study focuses on coupling ML architectures with porosity analysis in FDM components. The outcome is an ML-powered pipeline, capable of predicting porosity metrics from experimental batches without requiring extensive or costly testing.

## 2. Materials and Methods

### 2.1. Experimental 3D Printing Work

#### 2.1.1. Printing Using FDM

Porosity analysis was conducted using the following coupons:

- The 5 mm × 5 mm × 5 mm cubes: Referred to as ‘small’ in the rest of this study, it is the standard specimen size for defect assessment in metal additive manufacturing.
- The 10 mm × 10 mm × 10 mm cube: Referred to as ‘large’ in the rest of this study, it is printed under identical process parameters to evaluate dimensional scalability effects.

A MatterHackers Pulse XE printer (MatterHackers, Inc., Lake Forest, CA, USA) was used to fabricate the coupons, as it is cost-effective, user-friendly, and open-source. Table 1 summarizes the printer’s operational ranges for key variables. The printing material was a PolyLactic Acid (PLA) filament [40] from the MatterHackers Build Series, selected for its wide availability and reliability.

To isolate defect-induced porosity (as opposed to designed porosity), several variables were held constant due to their known or limited impact on print quality:

- Material: PLA was the only material explored through this investigation. It is widely used, recyclable, and does not require special storage conditions.
- Bed Temperature: The recommended value for PLA is 80 °C [40].
- Infill density: Fixed at 100%, since it is mainly for scaffolds and metamaterials with intentional voids.
- Printing orientation: Z-axis.

**Figure 2:**
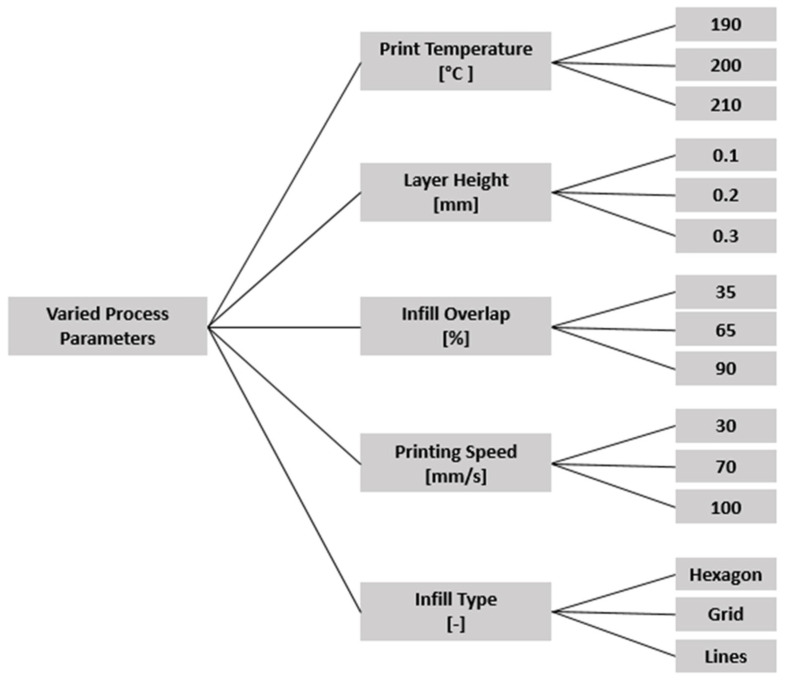
Varied process parameters for printing.

The remaining parameters—print temperature, layer height, infill overlap, speed, infill type—were varied within PLA-compatible ranges [41], and within the operational limits of the printer [42], as illustrated in Figure 2. of the 45 theoretically possible combinations, 11 were selected (Table 2) to balance the high cost of CT scanning with the need to sample a diverse region of the parameter space, focusing on combinations expected to produce a broad spectrum of porosity outcomes.

#### 2.1.2. Test Coupons

The coupons were modeled in CATIA V5 Release 31, sliced using the free MatterControl 3D Printing software, version 2.19, and then printed with the combinations of process parameters listed in Table 2. CT scanning was performed using a North Star Imaging X7000 system (North Star Imaging, Inc., Rogers, MN, USA). Initial scans at 10 µm (~4 h per scan) validated file processing workflows, and a 30 µm resolution was ultimately chosen as a compromise between cost and detail (1.5 h per scan). Given the defect tolerances in aerospace (hundreds to thousands of µm), this resolution was sufficient. The CT scan team conducted windowing and gray thresholding, and delivered 3D files readable by the efx-viewer software (Version 2.4.2.2. North Star Imaging, Inc., Rogers, MN, USA). Table 3 summarizes the main scanning features and values.

Each scan produced an efx 3D file, which was processed in efX-viewer to extract cross-sectional slices along the Z-axis (printing direction). Table 4 summarizes the number of slices per specimen set.

**Figure 3:**
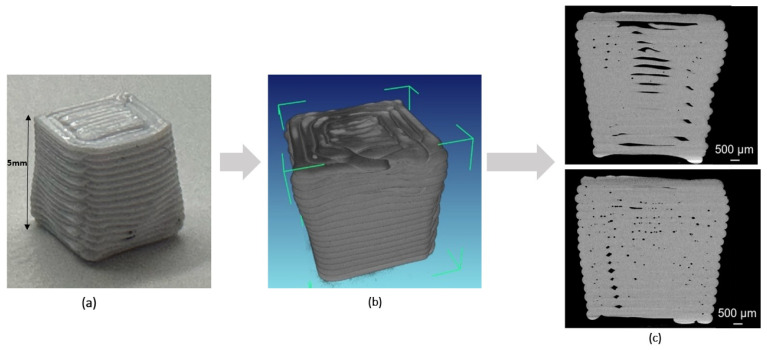
Process of image extraction: (**a**) 3D-printed cube, (**b**) digital file from the CT scan software, (**c**) generated 2D images of the cross-section along the z-axis.

Figure 3 summarizes the data pipeline: The CT scanner’s proprietary software efX-ct v2.3.6.0 reconstructs a 3D volume from 2D radiographic projections. The images were exported as 8-bit RGB PNGs, the available option in the efx-viewer, which are compatible with standard CNN libraries and still capture basic porosity defects accurately enough for the current investigation.

### 2.2. A Convolutional Neural Network for Image Classification

#### 2.2.1. Image Selection

**Figure 4:**
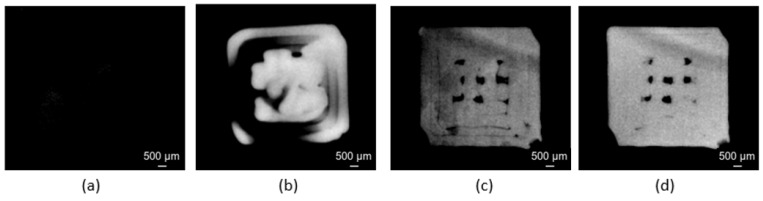
Examples of scan images: (**a**,**b**) are not exploitable. (**c**) presents defective pixels that mislead porosity analysis. (**d**) represents a suitable cross-section for porosity measurement.

The CT-scanned images varied in quality: some captured the background while others contained incomplete initial layer pixels (Figure 4a,b). Additional images exhibited defective pixels or uneven graying that skewed porosity calculations (Figure 4c). Only images showing complete cross-sections at the center with closed contours (Figure 4d) provided reliable porosity measurements.

**Figure 5:**
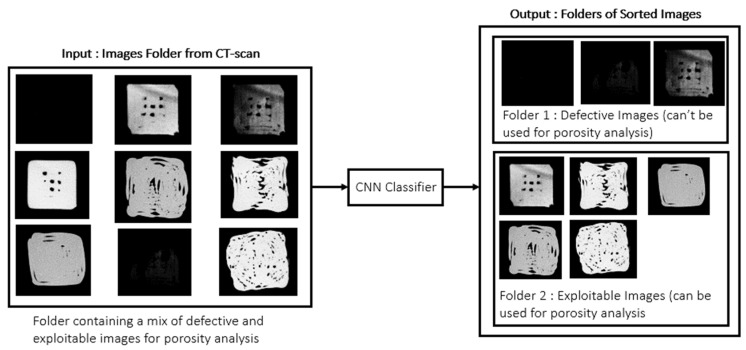
Principle of the CNN classifier.

Manual sorting of exploitable images is time-consuming and prone to error, often introducing outliers. Increasing the number or size of specimens would exacerbate this problem due to the larger number of slices requiring evaluation. To automate classification, a Convolutional Neural Network (CNN) [43] was selected for its robustness in image classification and ease of implementation and troubleshooting. Figure 5 illustrates the intended workflow of the classifier after training.

#### 2.2.2. Data Preparation

The dataset consists of 226 × 246 RGB PNG images, manually categorized into the following:

- Defective: Black, overly dark, or incomplete layers, where porosity analysis fails.
- Exploitable: Center of slice visible and well-formed, complete with closed contours, where porosity analysis succeeds.

To strengthen the CNN’s validation, all the slices from a given cube ID were kept entirely within either the training or testing set. This ensured a rigorous testing of the model’s generalization to unseen prints and prevented data leakage. Table 5 summarizes the number of data points per folder and scale. Most discarded slices primarily originated from the top and bottom layers.

#### 2.2.3. Model Construction

The CNN was implemented in Python 3.12.3 using TensorFlow 2.13 [44] and Keras 2.13 [45] (Google-supported, open-source ML libraries). The sequential architecture comprised convolutional and pooling layers for feature extraction, and fully connected layers for classification. The model was trained using the dataset split per cube ID.

To mitigate potential overfitting or learning issues arising from noise or the limited dataset size, k-fold cross-validation, an established statistical analysis method [46] and standard machine learning evaluation technique [47], was employed. This approach rigorously evaluates the model’s ability to generalize to independent data subsets.

The cross-validation procedure partitions the complete dataset into *n* equally sized subsets with comparable statistical characteristics. In each iteration, the CNN model is trained on *n* − 1 combined subsets and tested on the remaining holdout subset. This exhaustive process ensures that every data point is used exactly once for testing while contributing to *n* − 1 training cycles. Final performance metrics are computed by averaging results across all the folds, with the variance between folds serving as an indicator for model stability.

### 2.3. Extraction of Porosity Metrics

**Figure 6:**
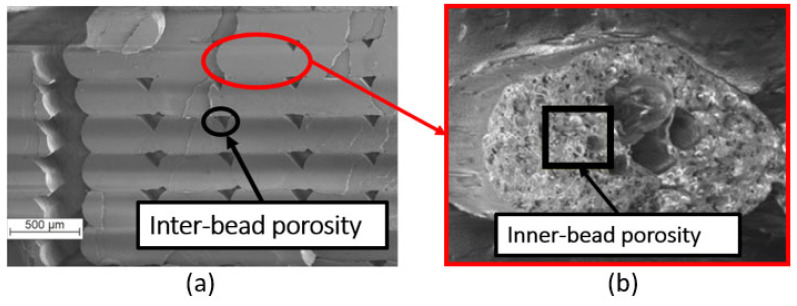
Main types of porosity encountered in FDM: (**a**) Inter-bead porosity [48], (**b**) Inner-bead porosity [49] (available via CC BY).

Porosity is defined as formation of voids or air pockets within a solid material. It compromises mechanical performance by creating stress concentrations at void boundaries. In FDM, porosity typically appears as follows:

- Inter-bead porosity: Gaps between deposited beads, aligned with the deposition direction (Figure 6a) [48].
- Inner-bead porosity: Voids within individual beads, generally negligible in homogeneous filaments compared to inter-bead defects (Figure 6b) [49].

Porosity arises when the molten bead exits the print head and pressure is no longer controlled or oriented. Rapid cooling affects viscosity, flowability, and diffusion, leading to the formation of voids.

Traditional porosity analysis, often integrated into CT reconstruction softwares, typically provides a single volumetric porosity percentage for an entire specimen. This scalar value accounts for all defects, including large-scale print failures such as warping, poor bed adhesion, or incomplete layers at the extremities. While useful for quantifying total void content, it offers no information on the spatial distribution or morphology of individual voids, which is critical for understanding mechanical performance and process-induced anomalies. Moreover, it does not provide layer-wise insights into porosity variations. In this study, porosity was calculated on a layer-by-layer basis using the extracted cross-section slices.

#### 2.3.1. Porosity Percentage

**Figure 7:**
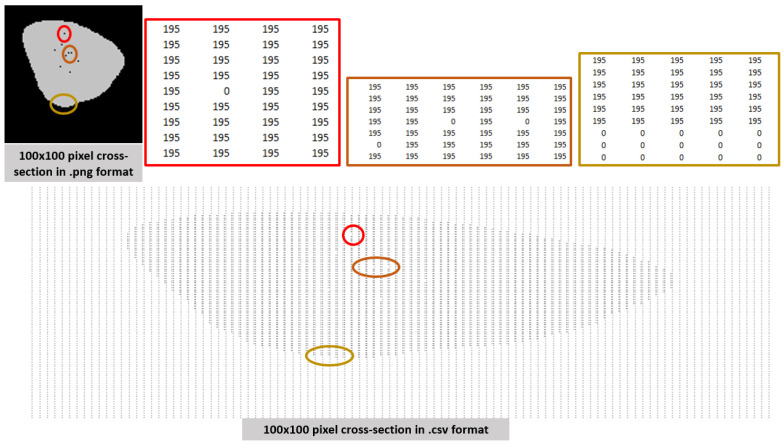
Breakdown of how the code manipulates an image through RGB values within a matrix on a spreadsheet.

Exploitable RGB images sorted by the CNN classifier contain closed-contour cross-sections. The following workflow was used to quantify porosity:

Pixel Analysis: The focus is on internal, isolated porosity defects. For initial segmentation, a global threshold of 50 (RGB scale) was applied to differentiate light pixels (material) from dark pixels (void) within the closed contour. The pixel values were exported to a matrix for quantification (Figure 7).

Sensitivity analysis: To evaluate threshold sensitivity, porosity percentages were recalculated using thresholds of 40 and 60.

**Figure 8:**
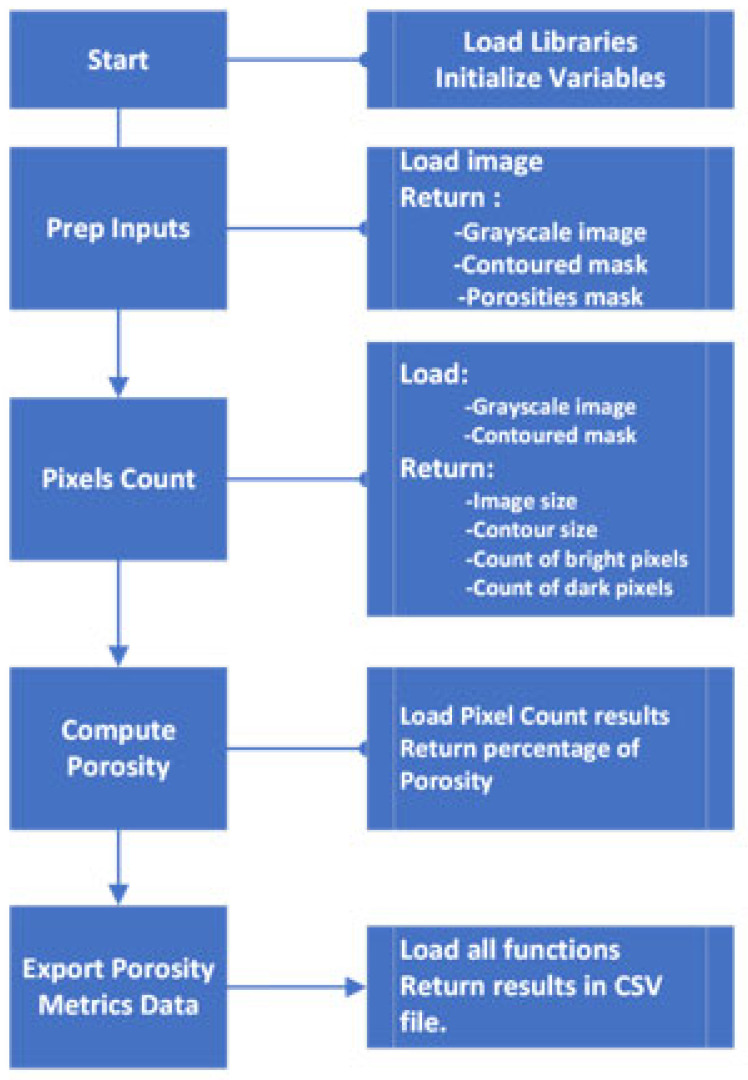
Porosity computation flowchart.

Porosity calculation: For each slice, porosity percentage was computed as the ratio of dark pixels to total pixels within the contour. The results were saved as a .csv file with image names, Z-heights, and porosity values according to the workflow described in Figure 8. The Z-height for each slice was calculated as the slice index generated by the efx-viewer multiplied by the slice spacing.

The code is available in the project’s GitHub repository (https://github.com/). The full results are in Appendix A [50].

#### 2.3.2. Porosity Distribution

As discussed in the introduction, studies on AM defects consistently emphasize the importance of correlating porosity distribution characteristics (including location and shape) with the resulting mechanical properties of printed components. To address this systematically, a streamlined algorithm was developed for porosity categorization. This methodology supports the current FDM analysis and establishes a foundation for future defect impact studies across both FDM and other AM processes.

Analysis of the exploitable images revealed two predominant porosity features, which can be effectively characterized using basic geometric shapes:

- Elliptical voids: These elongated voids typically appear stretched along a primary axis. They arise when two parallel extruded filaments fail to fuse completely during deposition, leaving an interstitial gap aligned with the bead deposition path.
- Circular voids: These approximately symmetrical voids appear with similar dimensions along all axes. They most often occur in central cross-sections due to insufficient cohesion between adjacent material regions, interlayer bonding deficiencies, or incomplete merging of filament paths.

**Figure 9:**
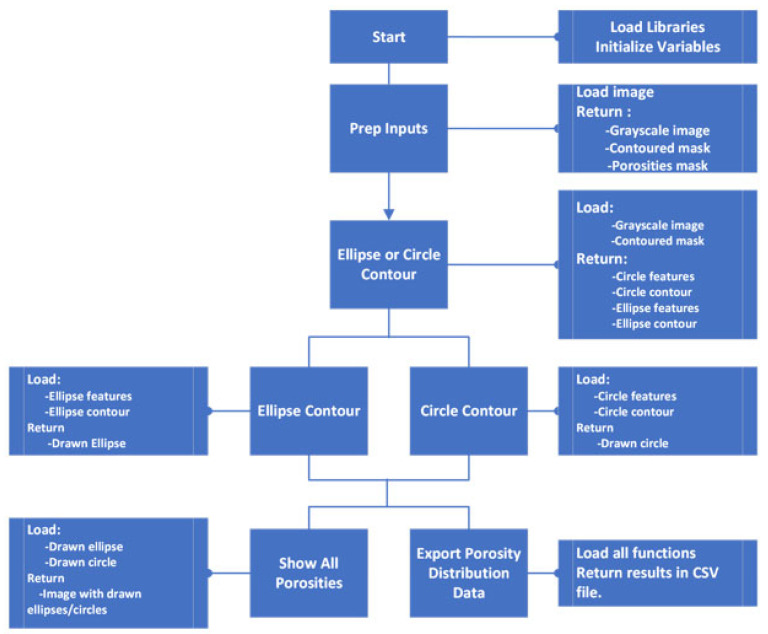
Porosity distribution flowchart.

To quantify these porosity distribution patterns systematically, a specialized image-processing algorithm was developed (Figure 9) with the following workflow:

- Image Preprocessing:
  - Converts the input RGB image to grayscale.
  - Applies intensity thresholding to isolate porosity features.
  - Performs contour extraction to precisely delineate void regions.
- Geometric Classification:
  - Employs two distinct mathematical approaches to approximate extracted contours: Covariance matrix analysis combined with eigenvalue decomposition, and Principal Component Analysis (PCA).
  - These methods collectively determine the major/minor axes and spatial orientation of each void.
  - Calculates aspect ratios to classify voids as either elliptical or circular
- Data Output and Visualization:
  - Generates comprehensive .csv files containing:
    ▪ Source image identifiers.
    ▪ Corresponding Z-height positions.
    ▪ Complete listings of all the classified porosity features with their geometric parameters.
  - Includes optional visualization modules that plot:
    ▪ Original extracted contours.
    ▪ Mathematically fitted ellipses and circles.
    ▪ Comparative overlays showing approximation accuracy.
  - Calculates IoU and plots results:
    ▪ Shows how well the elliptical and circular voids capture the morphology of the void.
    ▪ Plots indicators.

The algorithm processes entire directories of images in batch mode, ensuring consistent analysis while allowing inspection of individual images when required. This dual capability supports both large-scale statistical analysis and detailed examination of specific porosity formations.

#### 2.3.3. Repeatability Study

Three-dimensional printing is known to produce variable results for the same part, even under identical conditions. To quantify the intrinsic process variability under controlled conditions and establish a preliminary tolerance threshold for porosity variation, a repeatability study was conducted. Due to CT scanning cost and specimen availability, this analysis was limited to three specimens. The study provides initial insights while establishing the methodology. For better compliance with the statistical guidelines of aerospace standards, established sources such as NCAMP [51] recommend 10–15 specimens per batch for 3–5 batches. These guidelines are for mechanical characterization at the coupon level, however, and do not address defect quantification.

**Figure 10:**
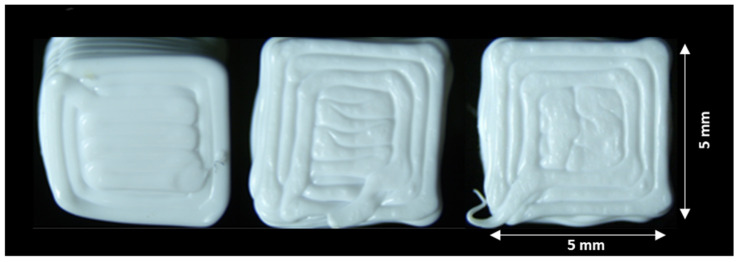
The 3D-printed cubes for repeatability study.

The three small cubes (see Figure 10) were printed using identical process parameters (see Table 6) under consistent ambient conditions. Each specimen underwent the following:

- Fabricated in separate print jobs (first/mid/last prints of the day).
- CT scanned individually at 30 µm resolution (1 h 15 min per scan).
- Reconstructed as 3D models using the scanner’s proprietary software.

### 2.4. A Multi-Layer Perceptron for Porosity Prediction

**Figure 11:**
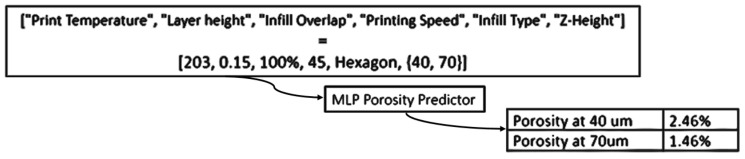
Principle of the MLP porosity predictor.

Predicting porosity based on independent variables with no established analytical correlation and using a finite dataset requires a robust regression approach. A multi-layer perceptron (MLP) was selected for its ability to model complex, non-linear relationships between printing parameters and porosity. Implemented using Keras [45], the feedforward neural network (Figure 11) is flexible and scalable for regression tasks.

#### 2.4.1. Data Preparation

Exploitable images filtered by the CNN classifier were processed to generate porosity percentages per Z-height, resulting in tabulated input–output pairs (Table 7).

Table 8 illustrates an example dataset combining printing parameters with the corresponding porosity measurements.

#### 2.4.2. Model Construction

The MLP was implemented using the Scikit-Learn library [52], one of the most widely adopted and documented ML libraries for Python. This MLP functions as a regression model, accepting the comprehensive set of printing variables (Table 6) as inputs and predicting porosity percentage as its single output. As a rule-of-thumb, the initial architecture is a simple one (2 hidden layers, 100 neurons), and complexity is progressively added as needed, based on performance evaluation and fine-tuning. The Adam optimizer was used with a default learning rate η = 0.001, known for stable convergence. Key aspects of the network initialization included the following:

- Data-Determined Parameters: Certain features were explicitly defined by the structure of the experimental data, such as the output layer—it consists of precisely one neuron, corresponding directly to the single output variable (porosity percentage).
- Empirically validated defaults: Other parameters were selected based on their established performance in similar regression applications:
  ▪ ReLU (Rectified Linear Unit) as the activation function, effective in regression tasks.
  ▪ Mean Squared Error (MSE) as the loss function, a standard for continuous value prediction tasks.
- Computationally optimized choices: The remaining configuration options were implemented to balance model performance with practical computational constraints:
  ▪ Adam optimizer (η = 0.001) for efficient gradient-based weight updates.
  ▪ A total of 1000 iterations specified as the initial training cycle count.
  ▪ Random seed fixed at 42 to ensure reproducibility.

This initialization ensures baseline configurations while allowing flexibility for hyperparameter tuning.

**Figure 12:**
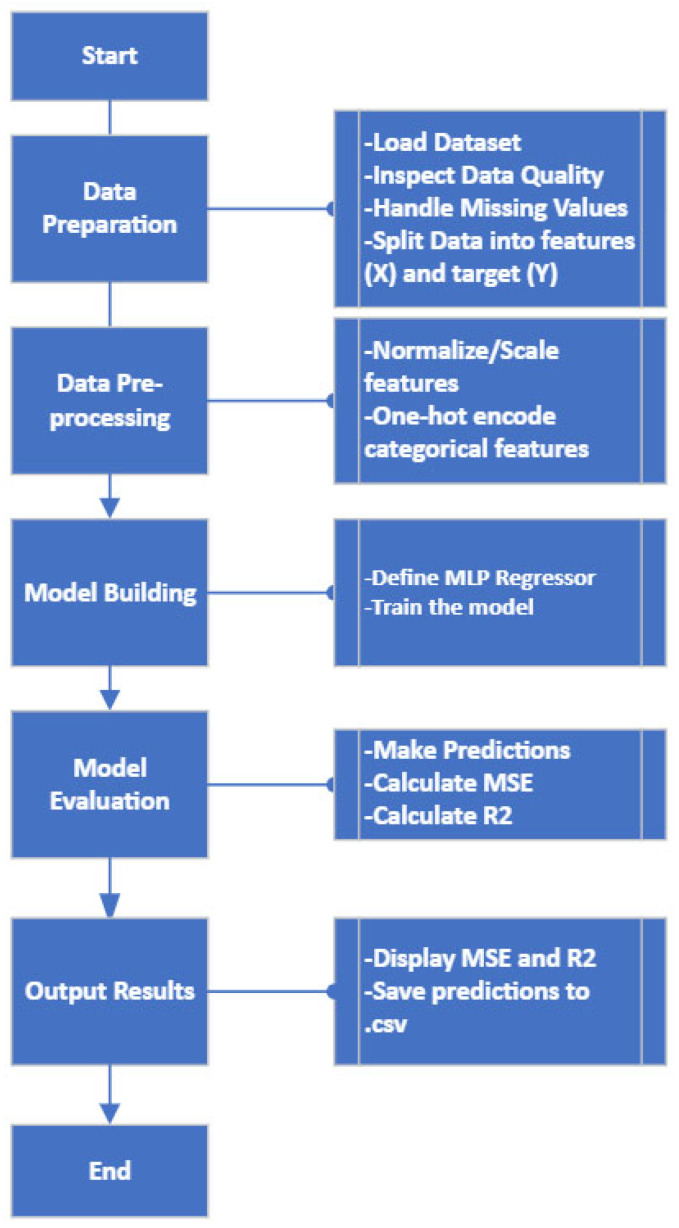
MLP Regressor model flowchart.

The model construction workflow is summarized in Figure 12:

- Preprocessing:
  ▪ Normalization/scaling of input features.
  ▪ Categorical encoding of string variables (e.g., infill type).
  ▪ Randomized training.
- Cross-Validation:
  ▪ k-fold partitioning to assess generalization.
  ▪ Iterative training on k-1 subsets with testing on held-out data.
  ▪ Averaged metrics across all folds.
- Bias Mitigation:
  ▪ Script-enforced random sampling.
  ▪ Validation against parameter variability (scale/type/range).

Consistent with the CNN dataset splitting strategy, MLP training data was organized by cube ID to prevent data leakage between training and testing sets, and k-fold cross-validation was used to further strengthen the training and testing.

### 2.5. Dimensional Scalability Investigation

To evaluate the effect of scaling dimensions on porosity defects and explore potential correlations across sizes, a dimensional scalability study was performed using data from the large cubes. The CNN classifier, trained on small cubes, was used to classify the large-cube slices. The same methodology for porosity analysis of the small cubes was replicated for the larger specimens to ensure consistency and comparability of results (Table 9).

The MLP initial configuration mirrored the one used for small cubes. Initial training iterations typically did not yield optimal results, necessitating careful fine-tuning to develop an accurate predictor while minimizing information loss. The final architecture of the MLP for large cubes is in Table A3.

## 3. Results

### 3.1. CNN Image Classifier

To automate classification of CT slices into ‘exploitable’ and ‘defective’ categories, a Convolutional Neural Network (CNN) was developed. The small-cube dataset was composed of approximately 2200 images, with 75% labeled as ‘exploitable’, and the remainder as ‘defective’. The CNN underwent several training iterations for hyperparameter fine-tuning and employed transfer learning to leverage pre-learned features for improved generalization. The VGG16 architecture [53], pre-trained on ImageNet [54], was used as the feature extraction backbone.

The classifier consisted of a global average pooling layer followed by two dense layers (128 and 1 neurons). To mitigate overfitting, a dropout layer (rate = 0.5) was applied. The model was compiled with the Adam optimizer and used binary cross-entropy as the loss function. Final architectural and training parameters are provided in Table A1.

**Figure A1:**
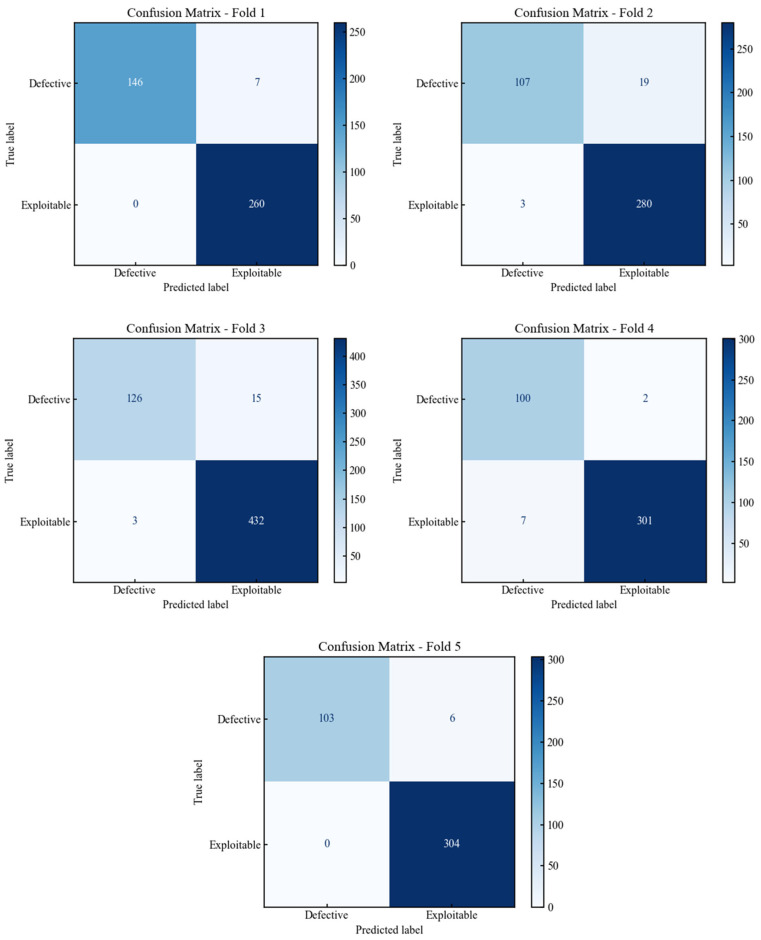
Confusion matrices of the CNN classifier over 5-fold grouped cross-validation for the small-cube dataset.

Four metrics were used to assess the performance of the CNN classifier: precision, recall, F1-score, and overall accuracy, averaged across five folds. The CNN demonstrated high and consistent performance, as detailed in Table 10, achieving an average accuracy of 97.2% ± 0.014 in the classification task. The ‘exploitable’ class showed slightly higher performance metrics than the ‘defective’ class. This was likely due to the larger number of ‘exploitable’ images. Confusion matrices for each fold (see Figure A1) further confirm the model’s classification capability across both classes.

The optimized classifier was subsequently applied to the large-cube images, yielding satisfactory results. Table 11 summarizes the classification outcomes: The overall average error in identifying defective images was 12.7% compared to 2.6% for the exploitable ones. As with the small-cube dataset, the large-cube dataset contains more exploitable than defective images, explaining the higher prediction accuracy for the exploitable class.

### 3.2. Porosity Analysis

**Figure 13:**
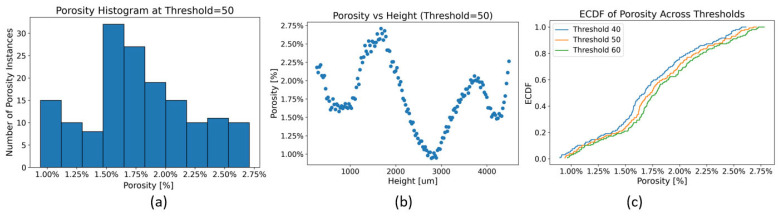
Example of data extracted from exploitable images of the CT-scanned coupon—small cube ID 1-190-100-35-30-Grid: (**a**) is a plot of the instances of porosity percentage, (**b**) is a scatter plot of the porosity percentage along the height, (**c**) is a graph of the ECDF of porosity across three thresholds.

Following the CNN classification, porosity analysis was performed on the exploitable images from both the small- and large-cube datasets. The developed scripts successfully quantified porosity percentages and spatial distributions per slice. As an example, Figure 13 summarizes the porosity characteristics for small cube ID 1-190-100-35-30-Grid. Most porosity instances fell within 1.5–1.75%, while the highest porosity values, exceeding 2.5%, occurred in the central layers and after the initial ones.

The sensitivity analysis confirmed the robustness of the porosity measurements: variations across threshold values were less than 0.12%, validating the segmentation approach and supporting the use of 50 as a global threshold value.

**Figure 14:**
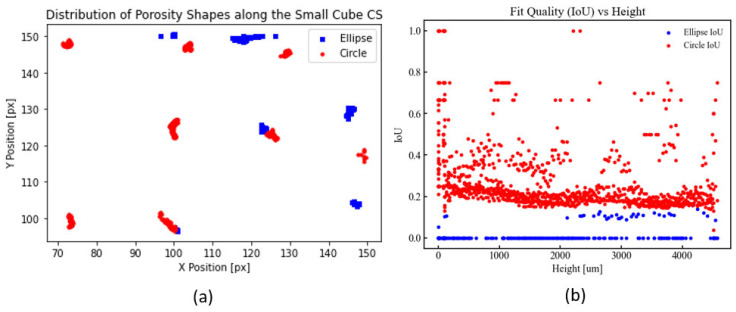
Example of data extracted from exploitable images of the CT-scanned coupon—small cube ID 1-190-100-35-30-Grid: (**a**) is a graph of the condensed layers of the specimen with the types and location of porosity, (**b**) is the fit quality of the idealized porosity shapes vs. height.

The visualization in Figure 14 allows qualitative assessment of porosity distributions and comparison of the void morphology to idealized elliptical or circular shapes. Quantitatively, the intersection over union (IoU) was used to evaluate model prediction performance. For small cube ID 1-190-100-35-30-Grid, the IoU ranged from within 0.2–0.4 for circular fits and was near 0.0 for elliptical fits, indicating that basic geometric approximations, while qualitatively capturing spatial distributions, poorly fit the void morphologies, particularly for the elliptical ones.

### 3.3. Investigation on Repeatability

Even under identical printing parameters and controlled environmental conditions, other uncontrolled process phenomena may influence porosity. To evaluate this, a repeatability framework was implemented using three small printed specimens produced under the same conditions.

The workflow generated images for each specimen, revealing two dominant porosity morphologies:

- Elliptical voids (Table 12 column 1): Elongated inter-bead voids resulting from insufficient cohesion between parallel deposited beads.
- Circular voids (Table 12 column 2): Localized spherical voids forming at bead boundaries due to incomplete interlayer fusion.

**Figure 15:**
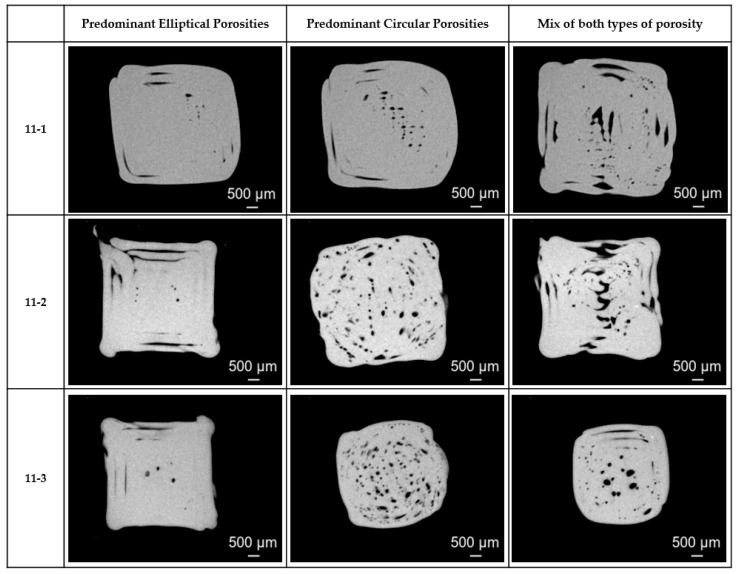
Example of cross-sections from specimens 11-1, 11-2, and 11-3.

Figure 15 illustrates cases of predominant elliptical porosity, predominant circular porosity, and mixed morphologies in the cross-sections of specimens 11-1, 11-2, and 11-3. After CNN-based image classification, the porosity analysis scripts processed all qualified cross-sections, producing a comprehensive .csv with porosity percentage and distribution. Table 12 summarizes the main findings from the data.

Key observations from the repeatability analysis include the following:

- Maximum porosity variation of 0.5% between specimens.
- Consistently low variance values, at 0.0001.
- Average porosity ranging from 1.85% to 2.36%.

**Figure 16:**
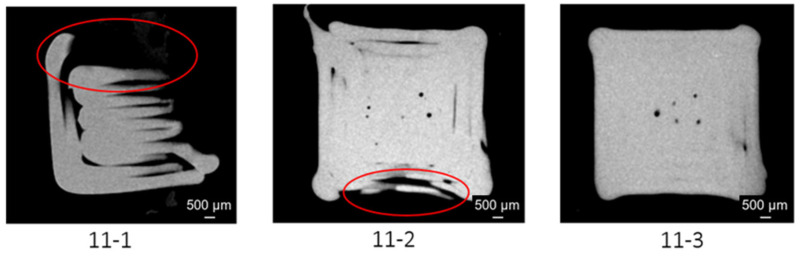
Cross-sections of the three specimens around 297 µm with red circle highliting problem areas: 11-1 exhibits an incomplete layer. 11-2 does not have a closed contour. 11-3 presents an ideal closed contour.

The initial height at which reliable porosity measurements could be obtained varied across specimens. Specimen 11-3 exhibited valid measurements as early as 297 μm, compared to 11-1 (540 μm) and 11-2 (432 μm). This discrepancy may be attributed to inconsistent initial layer deposition, as illustrated in Figure 16, which compares cross-sections at 297 μm:

- Specimen 11-1 exhibits incomplete layer formation.
- Specimen 11-2 lacks closed contours.
- Specimen 11-3 demonstrates ideal contour integrity.

Smoothing splines with cross-validated regularization were used to characterize the scattered porosity measurements across the build height. Model-to-model variability was quantified by evaluating the three independently fitted splines at a common set of N = 200 points along the height axis. The standard deviation σ(z) at each height z was calculated as the sample standard deviation of the three spline-predicted porosity values following Equation (1):

(1) $$\sigma (z)\;=\;\sqrt{\frac{\sum_{i=1}^{M}{\left(y_{i}\left(z\right)-\;\mu (z)\right)}^{2}}{M-1}}$$

where M = 3 is the number of samples, y_i_(z) is the spline-predicted porosity for sample i at height z, and μ(z) is the mean porosity across samples at that height. This approach generates an average porosity distribution along with a corresponding uncertainty zone (±1σ).

**Figure 17:**
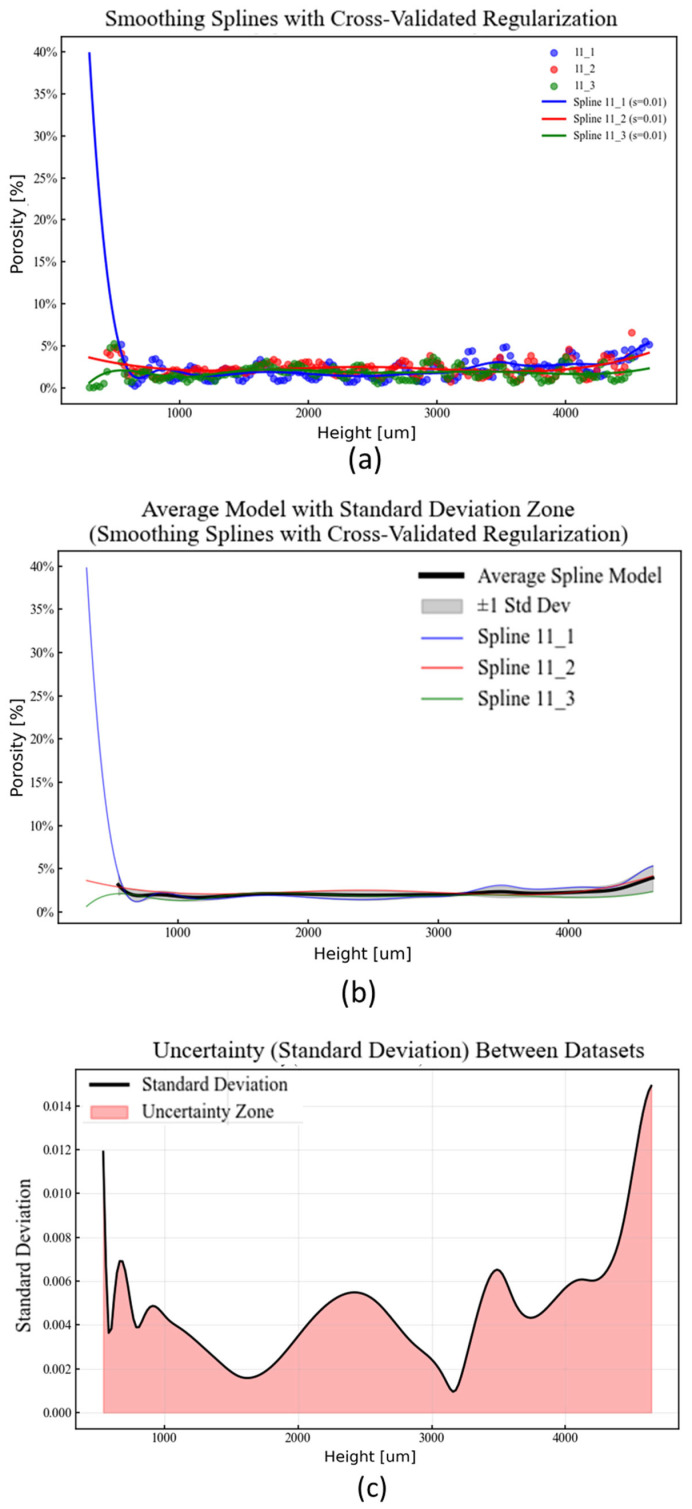
(**a**) Raw porosity data by height of the cubes 11-1, 11-2, and 11-3 with spline fits. (**b**) Mean model with uncertainty area. (**c**) Uncertainty profile across the height.

Figure 17 summarizes the repeatability study findings: Cross-validation identified an optimal smoothing parameter of s = 0.01 for all three datasets, indicating consistent data structure and noise levels. The spline fits achieved strong agreement with the uncertainty metrics, summarized in Table 13. The mean standard deviation was 0.47% with a maximum standard deviation of 1.49% porosity across the build height. The relative uncertainty, expressed as the ratio of the standard deviation to the mean porosity, was 21.55%. The pairwise RMS differences between the datasets were low, ranging from 0.0066 to 0.0087, with an average of 0.0074. These quantitative results indicate a high degree of consistency and repeatability in the porosity measurement process across different samples printed under identical conditions.

These patterns suggest a significant thermal influence during printing, particularly in interlayer cooling dynamics. While precise thermal control falls beyond this study’s scope, the data provide a quantitative basis for defining porosity variation thresholds.

Current aerospace validation protocols lack standardized frameworks for defect quantification. Existing specifications, such as MSFC-STD-3716 [55], mandate less than a few percent variation for critical additively manufactured components, while NCAMP guidelines [51] only address coupon levels and recommend statistical evaluation across 10–15 specimens per batch for 3–5 batches during mechanical characterization. Although no standard explicitly addresses defect distribution within a multiscale frame, the observed ±0.47% variation margin can inform machine learning models, enabling data-driven process optimization while maintaining quality control during production scaling.

### 3.4. Porosity Prediction

**Figure 18:**
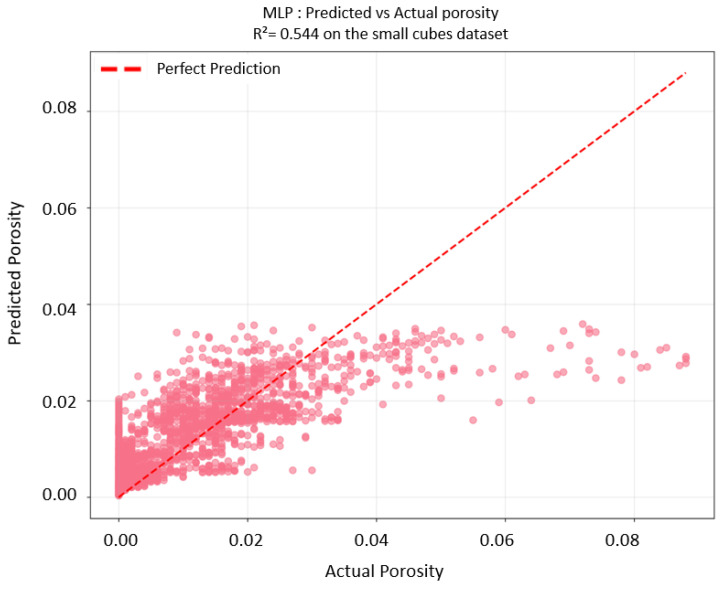
MLP prediction vs. actual porosity, trained on small-cube dataset.

The developed MLP model demonstrated the capability for porosity prediction. The final features of the small-cube MLP are available in the Table A2. The overall cross-validation MSE is 0.200 ± 0.135, and the R^2^ for the small-cube full dataset is 0.544. Figure 18 shows a comparison between the predicted porosity and the actual one.

**Figure 19:**
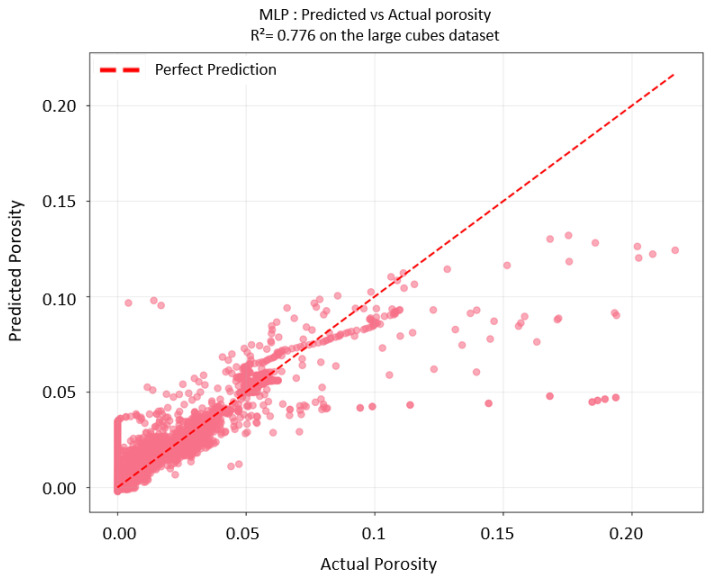
MLP prediction vs. actual porosity, trained on large-cube dataset.

The large-cube dataset was then used to train the MLP. Increased data points improved the results. The model achieved an overall cross-validation MSE is 0.125 ± 0.275, while the full dataset R^2^ increased to 0.776. The final architecture for the MLP trained on the large-cube dataset is reported in Table A3, with the updated prediction–observation comparison shown in Figure 19.

Overall, the finalized MLP framework trained on the large-cube dataset provides more accurate porosity percentage predictions across the parameter space of input printing variables and layer height coordinates. These results establish a robust computational foundation for quality assessment in additive manufacturing applications.

### 3.5. Scalability Investigation

The developed CNN and MLP models and the porosity analysis scripts demonstrated successful applicability to 10 mm × 10 mm × 10 mm specimens, confirming the pipeline’s adaptability for larger geometries and enabling rapid porosity analysis at increased scales.

To enable direct comparison of porosity profiles between cubes of different sizes (5 mm and 10 mm), and potentially different measurement resolutions, a two-step normalization strategy was followed. The physical height of each cube was min–max normalized to a dimensionless range of [0, 1], representing the relative position from the bottom (0) to the top (1) of each sample, according to Equation (2)

(2) $$Z_{norm}=\frac{Z-Z_{min}}{Z_{max}-Z_{min}}$$

Independently, each cube’s porosity values were min–max normalized to a [0, 1] range based on their respective observed minima and maxima, following Equation (3).

(3) $$P_{norm}=\frac{P-P_{min}}{P_{max}-P_{min}}$$

To ensure equal sampling resolution, porosity data from the larger cubes was linearly interpolated onto the normalized height coordinates of the smaller cube. This allowed robust, scale-invariant statistical comparison of porosity distribution morphologies.

**Figure 20:**
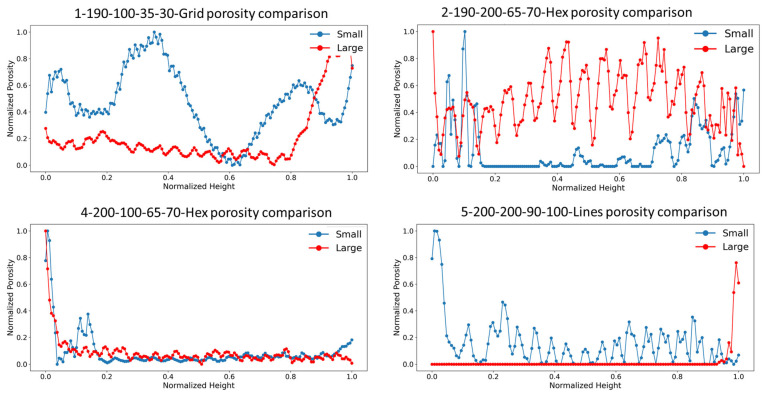
Example of normalized plots of porosity comparison between small and large cubes.

Figure 20 illustrates the normalized porosity profiles for representative small and large cube IDs. No systematic trend was observed linking porosity behavior between the small and large specimens. Instead, each process-parameter combination produced distinct porosity distributions, further emphasizing process sensitivity.

Statistical analysis, summarized in Table 14 and Table 15, further highlights this lack of scalability. The average correlation coefficient was only 0.34, while the consistently extreme T-statistics (avg. 4.52) and *p*-values averaging 0.15 further confirm that differences between small- and large-cube porosity distributions are statistically significant and not due to random variation. Moreover, small cubes exhibited 69% higher mean porosity, quantitatively demonstrating a pronounced scale effect in defects within the FDM-printed components.

Considering the lack of standardized thresholds for porosity distribution in current aerospace and AM standards, the observed scale effect (small cubes having ~69% higher mean porosity) and repeatability margin (±0.47%) offer an empirical basis for defining component-specific tolerances. These could align with existing reporting and inspection standards (such as FAA AC 33.15-3 [56] and ASTM F2971 [57]) to strengthen qualification protocols, especially as AM parts are scaled up.

## 4. Conclusions and Future Prospects

This work established a machine learning framework for porosity analysis and prediction in FDM, demonstrating that data-driven approaches can reduce trial-and-error costs while addressing scale-dependent defect formation. The pipeline, initially developed for small cubes and later applied to larger cubes, proved adaptable to new data and represents a first step toward a digital twin for porosity analysis and prediction, with quantified uncertainty for every model.

### 4.1. Key Findings

The investigation yielded five principal outcomes:

- Porosity Analysis: Development of a modular algorithmic framework capable of layer-specific porosity quantification (percentage, void location, and classification into ellipse- or circle-like geometries), offering insight into the anisotropic and heterogeneous nature of FDM components.
- Repeatability study: Establishment of a methodology to assess process variability under identical parameters, showing consistent porosity measurements within ±0.47% variation bounds.
- Porosity Prediction: Implementation of convolutional and perceptron networks, achieving over 90% classification accuracy for CNN, an R^2^ of 54.4% for MLP on small cubes, increasing to 77.6% on the large ones.
- Dimensional Scalability Effects: Component scale significantly influences porosity characteristics. Despite identical printing conditions, 10 mm × 10 mm × 10 mm specimens exhibited distribution patterns distinct from their 5 mm × 5 mm × 5 mm counterparts. Statistical analysis confirmed systematic differences, with an average correlation coefficient of 0.34, consistently extreme T-statistics (avg. 4.52) and *p*-values (avg. 0.15), thus confirming porosity distributions between small and large cubes are fundamentally different.
- Industrial Relevance: Development of practical predictive tools to reduce empirical parameter optimization and enable model transfer to higher-cost AM processes.

### 4.2. Limitations of the Present Research

The following work exhibits four primary constraints:

- Dataset Diversity: The multiscale analysis was restricted to cubic geometries at micro/meso-scales, limiting direct extrapolation to full-scale components.
- Vertical porosity distribution: Exported slices were along the vertical direction. Including additional orthogonal orientations would strengthen porosity distribution assessment.
- Model Complexity: The CNN-MLP framework may not capture all porosity formation mechanisms under non-standard printing conditions or atypical defect morphologies.
- Validation scope: The repeatability assessment would benefit from a larger sample size to strengthen variation quantifications.
- Material Focus: While PLA was used to establish a cost-effective methodological pipeline, the framework is material-agnostic and should be validated on other polymers such as ABS and Nylon.

### 4.3. Recommendations for Future Work

- Expanding datasets to include more materials, geometries, and machine parameters, while incorporating geometric descriptors (e.g., volume, surface-area-to-volume ratio, and critical distances from edges) to improve model robustness and generalizability.
- Enhance ML capabilities to predict void morphologies and their distribution along the build height.
- Develop data-driven models to explore parameter interactions and establish phenomenological laws for cross-scale porosity prediction.
- Extend the framework to PBF processes, where datasets are more expensive to obtain, allowing exploration of the assumption that low-cost AM data can inform defect prediction in high-cost processes.

### 4.4. Industry Impact

Based on the research conducted so far, the following recommendations are proposed:

- Parameter Optimization: Applying identified process windows to minimize porosity, with validation for specific material-machine combinations.
- ML System Integration: Deployment of the trained models as quality control modules within existing AM workflow softwares for continuous monitoring.
- Knowledge Transfer: Testing the transfer of porosity prediction between different FDM machines.

Impact would then be measured in the following areas:

- Quality Assurance: The integration of ML models provides real-time predictive insights, allowing proactive parameter adjustments to enhance part reliability.
- Cost Efficiency: By replacing trial-and-error methods with data-driven approaches, production costs and time can be minimized.
- Methodological Innovation: The hybrid ML framework establishes a scalable template for quality assessment across AM technologies and materials.
- Cross-Sectoral Applicability: The findings demonstrated capabilities directly addressing stringent reliability requirements in aerospace and medical device manufacturing.

## Figures and Tables

**Table 1:** Ranges of main 3D printing properties for Pulse XE.

Parameter	Range
Infill Density [%]	0–100%
Printing Orientation	X–Y–Z–XY–YZ–XZ
Bed Temperature [°C]	80 °C–115 °C ± 5 °C
Material	PLA–ABS–Nylon
Print Temperature [°C]	190–290 °C ± 5 °C
Layer Height [µm]	100–300
Infill Overlap [%]	0–100%
Printing Speed [mm/s]	0–100
Infill Type	Grid–Gyroid–Concentric–Hexagon–Triangle–Lines

**Table 2:** Summary of combinations of process parameters for coupon printing.

Designation	Print T	Layer Height	Infill Overlap	Printing Speed	Infill Type
-	[°C]	[µm]	[%]	[mm/s]	-
1-190-100-35-30-Grid	190	100	35	30	Grid
2-190-200-65-70-Hexagon	190	200	65	70	Hexagon
3-190-300-90-100-Lines	190	300	90	100	Lines
4-200-100-65-70-Hexagon	200	100	65	70	Hexagon
5-200-200-90-100-Lines	200	200	90	100	Lines
6-200-300-35-30-Grid	200	300	35	30	Grid
7-210-100-90-100-Lines	210	100	90	100	Lines
8-210-200-35-30-Grid	210	200	35	30	Grid
9-210-300-65-70-Hexagon	210	300	65	70	Hexagon
10-210-300-30-35-Grid	210	300	30	35	Grid
11-210-300-90-100-Lines *	210	300	90	100	Lines

* two additional cubes with the same combination of printing parameters were manufactured. They are part of the repeatability study.

**Table 3:** Summary of combinations of scanning features for small and large coupons.

Scanning Features	Value
Reconstructed pixel pitch	0.02991 mm
Slice spacing	29.91 µm
Pixel size X, Y, Z	29.91 µm/pixel
Voxel size	29.91 µm × 29.91 µm × 29.91 µm

**Table 4:** Number of slices extracted from the 3D file per specimen.

Designation	Number of Slices [Small]	Number of Slices [Large]
1-190-100-35-30-Grid	215	382
2-190-200-65-70-Hexagon	216	376
3-190-300-90-100-Lines	211	397
4-200-100-65-70-Hexagon	229	369
5-200-200-90-100-Lines	197	384
6-200-300-35-30-Grid	181	392
7-210-100-90-100-Lines	211	372
8-210-200-35-30-Grid	198	416
9-210-300-65-70-Hexagon	213	371
10-210-300-30-35-Grid	213	375
11-210-300-90-100-Lines	198	367

**Table 5:** Number of slices for small coupons per folder.

Designation	N. of Slices	N. of Exploitable Slices	N. of Defective Slices	Percentage of Defective Slices
-	-	-	-	[%]
1-190-100-35-30-Grid	215	162	53	24.65%
2-190-200-65-70-Hexagon	216	157	59	27.31%
3-190-300-90-100-Lines	211	162	49	23.22%
4-200-100-65-70-Hexagon	229	160	69	30.13%
5-200-200-90-100-Lines	197	127	70	35.53%
6-200-300-35-30-Grid	181	162	19	10.50%
7-210-100-90-100-Lines	211	161	50	23.70%
8-210-200-35-30-Grid	198	157	41	20.71%
9-210-300-65-70-Hexagon	213	147	66	30.99%
10-210-300-30-35-Grid	213	160	53	24.88%
11-210-300-90-100-Lines	198	153	45	22.73%

**Table 6:** Printing variables and dimensional measurements for repeatability specimens.

ID	-	11-1	11-2	11-3
Print Temp	[°C]	210		
Layer height	[µm]	300		
Infill Overlap	[%]	90		
Printing Speed	[mm/s]	100		
Infill Type	-	Lines		
x-length *	[mm]	5.65	5.6	5.29
y-length *	[mm]	5.62	5.77	5.62
z-length *	[mm]	5.4	5.13	5.22
Printing Time	-	Mid-Print of the day	1st print of the day	Last print of the day

* dimensions were measured with a Clockwise Tools (Valencia, CA, USA) IP54 caliper of ± 0.03 mm accuracy.

**Table 7:** Neural network input/output structure.

Input Data	Output
Print Temperature Layer Height Infill Overlap Printing Speed Infill Type Z-Height	Porosity

**Table 8:** Example of dataset (small cube) used for porosity prediction.

Cube ID	Input						Output
	Print Temperature	Layer Height	Infill Overlap	Printing Speed	Infill Type	Z-Height	Porosity
-	[°C]	[µm]	[%]	[mm/s]	-	[µm]	[%]
1	190	100	35	30	Grid	135	1.64
1	190	100	35	30	Grid	216	1.92
2	190	200	65	70	Hexagon	3564	0.01
2	190	200	65	70	Hexagon	3591	0.03
10	210	300	30	35	Grid	4536	4.27
10	210	300	30	35	Grid	4563	4.64
7	210	100	90	100	Lines	3780	0.01
7	210	100	90	100	Lines	3807	0.01
6	200	300	35	30	Grid	3321	2.03
6	200	300	35	30	Grid	3348	2.26

**Table 9:** Example of dataset from the large cubes used for porosity prediction.

Cube ID	Input						Output
	Print Temperature	Layer Height	Infill Overlap	Printing Speed	Infill Type	Z-Height	Porosity
-	[°C]	[µm]	[%]	[mm/s]	-	[µm]	[%]
1	190	100	35	30	Grid	870	6.38
2	190	200	65	70	Hexagon	3540	1.05
2	190	200	65	70	Hexagon	3570	1.17
10	210	300	30	35	Grid	6960	0.95
10	210	300	30	35	Grid	6990	1.36
7	210	100	90	100	Lines	7410	0.00
7	210	100	90	100	Lines	7440	0.00
6	200	300	35	30	Grid	2580	1.84
6	200	300	35	30	Grid	2610	2.64

**Table 10:** CNN classifier performance metrics from 5-fold grouped cross-validation.

Performance Metric	Defective Class	Exploitable Class	Total
	Mean ± Std	Mean ± Std	Mean ± Std
Precision	0.977 ± 0.026	0.970 ± 0.021	-
Recall	0.925 ± 0.052	0.992 ± 0.009	-
F1-Score	0.949 ± 0.026	0.981 ± 0.010	-
Accuracy	-	-	0.972 ± 0.014

**Table 11:** Number of datapoints for large cubes per folder.

Designation	Predicted Exploitable	Actual Exploitable Slices	Predicted Defective	Actual Defective
1-190-100-35-30-Grid	328	325	54	57
2-190-200-65-70-Hexagon	325	319	51	57
3-190-300-90-100-Lines	325	311	72	86
4-200-100-65-70-Hexagon	328	322	41	47
5-200-200-90-100-Lines	330	326	54	58
6-200-300-35-30-Grid	320	318	72	74
7-210-100-90-100-Lines	329	319	43	53
8-210-200-35-30-Grid	327	325	89	91
9-210-300-65-70-Hexagon	317	303	54	68
10-210-300-30-35-Grid	326	322	49	53
11-210-300-90-100-Lines	324	300	43	67

**Table 12:** Overview of general features from the cube data.

Cube ID	11-1	11-2	11-3
Number of generated images	198	205	228
Exploitable images	153	152	157
First height for porosity from exploitable cross-section [µm]	540	432	297
Last height for porosity from exploitable cross-section [µm]	4644	4509	4509
Min porosity [%]	0.33%	0.77%	0.12%
Max porosity [%]	5.55%	6.63%	5.30%
Avg. porosity [%]	2.22%	2.36%	1.85%
Range	0.0522	0.0586	0.0518
Variance	0.0001	0.0001	0.0001

**Table 13:** Uncertainty metrics calculated over 11-1, 11-2, and 11-3.

Metrics	Value
Mean standard deviation	0.0047
Maximum standard deviation	0.0149
Relative uncertainty (std/mean)	21.55%
RMS difference between datasets 1 and 2	0.0069
RMS difference between datasets 1 and 3	0.0087
RMS difference between datasets 2 and 3	0.0066
Average pairwise difference	0.0074

**Table 14:** Statistical analysis of porosity sets (mean, standard deviation, and correlation).

Specimen	N of Data Points	Small Cubes		Large Cubes		Comparison		Correlation *
		Mean	Standard Deviation	Mean	Standard Deviation	Mean	Standard Deviation	
1-190-100-35-30-G	162	0.48	0.25	0.21	0.23	0.56	0.08	−0.01
2-190-200-65-70-H	157	0.12	0.18	0.49	0.21	−3.08	−0.17	−0.18
3-190-300-90-100-L	161	0.29	0.25	0.06	0.13	0.79	0.48	0.08
4-200-100-65-70-H	160	0.09	0.14	0.08	0.11	0.11	0.21	0.80
5-200-200-90-100-L	127	0.15	0.19	0.02	0.10	0.87	0.47	−0.12
6-200-300-35-30-G	162	0.18	0.19	0.22	0.17	−0.22	0.11	0.61
7-210-100-90-100-L	161	0.10	0.26	0.03	0.14	0.70	0.46	0.06
8-210-200-35-30-G	157	0.19	0.17	0.10	0.15	0.47	0.12	0.58
9-210-300-65-70-H	160	0.26	0.19	0.11	0.17	0.58	0.11	−0.06
10-210-300-30-35-G	147	0.24	0.17	0.09	0.15	0.63	0.12	0.68
11-210-300-90-100-L	153	0.36	0.22	0.06	0.16	0.83	0.27	0.43
Min Value	127	0.09	0.14	0.02	0.10	−3.08	−0.17	−0.18
Max Value	162	0.48	0.26	0.49	0.23	0.87	0.48	0.80
Average Value	155.18	0.22	0.20	0.13	0.16	0.20	0.21	0.34

* negative values indicate inverse relationships.

**Table 15:** Statistical analysis of porosity datasets (T-statistic, *p*-value, and area).

Specimen	N of Datapoints	T-Statistic	*p*-Value	Area Between Curves
190-100-35-30-Grid	162	9.758	0.000	0.36
190-200-65-70-Hex	157	−16.701	0.000	0.42
190-300-90-100-Lines	161	9.971	0.000	0.27
200-100-65-70-Hex	160	0.210	0.834	0.05
200-200-90-100-Lines	127	7.230	0.000	0.16
200-300-35-30-Grid	162	−2.203	0.028	0.13
210-100-90-100-Lines	161	3.257	0.001	0.11
210-200-35-30-Grid	157	5.274	0.000	0.12
210-300-65-70-Hex	160	7.576	0.000	0.23
210-300-30-35-Grid	147	8.473	0.000	0.17
210-300-90-100-Lines	153	13.303	0.000	0.30
Min Value	127	−16.70	0.00	0.05
Max Value	162	13.30	0.83	0.42
Average Value	155.18	4.52	0.15	0.18

## Data Availability

The raw data supporting the conclusions of this article will be made available by the authors on request.

## Abbreviations

The following abbreviations are used in this manuscript:

BES	Bald Eagle Search
PSO	Particle Swarm Optimization
ML	Machine Learning
AM	Additive Manufacturing
FDM	Fused Deposition Modeling
CNN	Convolutional Neural Network
MLP	Multi-Layer Perceptron
MSE	Mean Squared Error
PCA	Principal Component Analysis
ReLU	Rectified Linear Unit

## Appendix A

**Table A1:** Final CNN architecture and training parameters.

Feature	Value	Comment
Classes	2 (Defective, Exploitable)	Binary classification task
CNN Network’s Type	Sequential	Linear layer stack for straightforward image processing
Convolutional Layers	3	Initial depth for feature extraction
Pooling Layers	3	Downsampling to reduce spatial dimensions
Fully Connected Layers	2	Final layers for classification
Batch size	10	Balances memory usage and gradient stability
Epoch	15	Initial training cycles
Activation Function 1	ReLU	For non-linearity
Activation Function 2	Sigmoid	For binary probabilities.
Dataset size	2282	Number of images

**Table A2:** Final MLP configuration for small cubes.

Network’s Features	Value	Comments
hidden_layer_sizes	(200, 100)	Two hidden layers with 200 and 100 neurons, respectively
activation	ReLU	Best candidate for empirical success in regression
output Layer	One neuron	Single output (porosity)
optimizer	Adam	Stochastic gradient-based optimization
learning_rate_init	0.00597	Initial learning rate for Adam optimizer
alpha	0.001835	L2 Regularization
batch_size	32	Batch size for stochastic optimization
beta_1	0.99	Exponential decay rate for first moment estimates
beta_2	0.999	Exponential decay rate for second moment estimates
early_stopping	True	Validation-based early stopping
n_iter_no_change	20	Number of iterations with no improvement to wait before stopping
max_iter	1000	Maximum number of iterations
Random seed	42	Reproducibility
Total number of data	1708	Full dataset
Epoch	100	Total number of iterations of all the training data in one cycle

**Table A3:** Final MLP configuration for large cubes.

Network’s Features	Value	Comments
hidden_layer_sizes	(200, 100)	Two hidden layers with 200 and 100 neurons, respectively
activation	ReLU	Best candidate for empirical success in regression
output Layer	One neuron	Single output (porosity)
optimizer	Adam	Stochastic gradient-based optimization
learning_rate_init	0.0066	Initial learning rate for Adam optimizer
alpha	0.0069	L2 Regularization
batch_size	32	Batch size for stochastic optimization
beta_1	0.95	Exponential decay rate for first moment estimates
beta_2	0.999	Exponential decay rate for second moment estimates
early_stopping	True	Validation-based early stopping
n_iter_no_change	20	Number of iterations with no improvement to wait before stopping
max_iter	1000	Maximum number of iterations
Random seed	42	Reproducibility
Total number of data	3745	Full dataset
Epoch	100	Total number of iterations of all the training data in one cycle
